# Alirocumab Plus Cemiplimab in Immunorefractory NSCLC: A Single Arm Phase 2 Study

**DOI:** 10.21203/rs.3.rs-10011569/v1

**Published:** 2026-06-24

**Authors:** Eziafa Oduah, Jhanelle Gray, Thomas Stinchcombe, Steven Wolf, Xiaodi Qin, Abbie Ireland, Joel Rivera-Concepcion, Jeffrey Clarke, Jeffrey Crawford, Laura Alder, Cameron Oswalt, Alan Chen, Liliana Lyniv, Aamna Abbasi, Andreas Saltos, Sin-Ho Jung, Kouros Owzar, Neal Ready, Trudy Oliver, Scott Antonia

**Affiliations:** Duke Cancer Institute, Duke University School of Medicine; Moffitt Cancer Center; Duke Cancer Institute; Duke Cancer Institute, Duke University; Duke Cancer Institute, Duke University School of Medicine; Duke Cancer Institute, Duke University Health System; Duke Cancer Institute; Duke Cancer Institute, Duke university School of Medicine; Duke Cancer Institute, Duke university School of Medicine; Duke Cancer Institute, Duke university School of Medicine; Duke Cancer Institute, Duke university School of Medicine; Duke Cancer Institute, Duke university School of Medicine; Duke Cancer Institute, Duke University; H. Lee Moffitt Cancer Center and Research Institute; Duke University; Duke University; Duke Cancer Institute, Duke University; Duke Cancer Institute, Duke University; Moffitt Cancer Center

**Keywords:** PCSK9, Immunotherapy, immune checkpoint blockade, immunotherapy resistance, NSCLC

## Abstract

In preclinical models, PCSK9 mediates cancer immunotherapy resistance and may serve as a novel immuno-inhibitory target. Herein, we report the results from a multi-center, single arm, phase II study evaluating the clinical activity and safety of the PCSK9 inhibitor alirocumab, in combination with the anti-PD1 antibody cemiplimab, in non-small cell lung cancer (NSCLC) patients with disease progression after previous immune checkpoint blockade. The primary endpoint was objective response rate (ORR). Secondary endpoints were progression-free survival (PFS), overall survival (OS), duration of response (DOR), disease control rate (DCR) and safety, and an exploratory objective was to analyze potential biomarkers of response. Sixty patients were enrolled, and 58 were evaluable for ORR. The ORR was 14.78% (90% CI, 5.30, 25.43), the median PFS was 2.5 months (95% CI, 1.5–3), and the median OS was 7.3 months (95% CI, 5.4 – 12.3). The most common treatment related adverse events (all grades) were anemia and fatigue. Grade 3 adverse events occurred in seven (12%) patients and were anemia, Guillain-Barre Syndrome and elevated amino transferase. There were no treated related adverse events of grade 4 or greater in the study population. Biomarker analysis identified superior outcomes in NSCLC harboring PIK3CA, PTEN, or AKT1 alterations. The ORR in patients with PIK3CA, PTEN, or AKT1 alterations (n=17) was 29.4% (95% CI, 10.3% - 56.0%). No objective responses were observed in the absence of PIK3CA, PTEN or AKT1 alterations (n=39). The presence of PIK3CA, PTEN or AKT1 alterations was significantly associated with response, p 0.0032 (two-sided, Fisher’s exact test). Further translational studies revealed the impact of PIK3CA, PTEN or AKT1 alterations on intratumoral PCSK9, providing a biological rationale for the pattern of response and clinical benefit. These findings provide clinical proof-of-principle that PCSK9 inhibition can overcome immunotherapy resistance in a subset of patients and suggest that PIK3CA/PTEN/AKT1 pathway plays a significant role in PCSK9 mediated immune evasion and could be a biomarker of response. These findings warrant further investigation in larger confirmatory studies.

Activation of the anti-tumor immune response by immune checkpoint inhibitors (ICI) revolutionized the treatment of non-small cell lung cancer (NSCLC) without actionable genomic alterations and improved clinical outcomes in frontline treatment regimens^1–3^. Despite this, the efficacy of ICI therapy is limited by primary and secondary resistance. Fewer than 20% of patients with metastatic NSCLC treated with ICI achieve durable response and are alive at five years^4–7^. These statistics underscore the pressing need for strategies that overcome resistance and enhance responses to ICI therapy and thereby improve survival.

For patients with NSCLC lacking actionable genomic alterations who progress after first-line chemo-immunotherapy, standard-of-care therapy options have limited clinical efficacy and are associated with significant toxicities. Treatment with docetaxel with or without ramucirumab has a response rate of approximately 7–20%, median progression-free survival of approximately three to four months, and median overall survival of 7.5–10 months^8–10^. Although several novel therapies have shown preliminary activity, none have produced a definitive overall survival improvement compared with docetaxel^5,11–15^. Consequently, there remains a critical unmet clinical need for therapeutic options that are safe and efficacious in overcoming immunotherapy resistance, an area of active investigation.

Proprotein convertase subtilisin/kexin type 9 (PCSK9) encoded by the *PCSK9* gene on chromosome 1p32.3 is primarily known for its role cholesterol metabolism,^16,17^ and has recently emerged as an inhibitory immunoregulator within the tumor microenvironment. The mechanisms underlying PCSK9-mediated immune evasion have been described in preclinical studies^18–21^, including through MHC I degradation, increased myeloid derived suppressor cells (MDSCs), decreased MHC II expression, and decreased recycling of T cell receptor complexes. In murine models, PCSK9 inhibition synergized with immune checkpoint inhibitors to increase intratumoral CD8^+^ T cell infiltration and activity^22–23^. Limited retrospective clinical evidence has associated higher PCSK9 levels with poor outcomes in immunotherapy-treated NSCLC patients^24,25^, More recently a retrospective analysis in ICI-treated cancer patients indicated that concomitant treatment with a PCSK9 inhibitor for cholesterol management was associated with improved survival in cancer patients undergoing immunotherapy^26^. However, to our knowledge, the clinical efficacy of PCSK9 inhibition to overcome immunotherapy resistance in a prospective clinical trial has not been reported.

To investigate the efficacy of PCSK9 inhibition to overcome immunotherapy resistance in patients with NSCLC, we initiated a clinical trial evaluating the efficacy and safety of PCSK9 inhibition in combination with immune checkpoint blockade. This multicenter, open label, single-arm phase II study tested the anti-PCSK9 monoclonal antibody alirocumab in combination with the anti-PD1 antibody cemiplimab in patients with metastatic NSCLC whose disease progressed on prior checkpoint inhibitor therapy. In parallel, we incorporated translational analyses to explore molecular determinants of response. Here, we examine the clinical activity of PCSK9 inhibition and investigate the role of alterations in the PIK3CA/PTEN/AKT1 axis in shaping tumor-derived PCSK9 expression and therapeutic sensitivity, providing a biological framework for biomarker selection in immunotherapy-refractory NSCLC.

## Results

### Patients and Treatments

Between May 2023 and September 2025, 60 patients with advanced/metastatic previously immunotherapy-resistant NSCLC were enrolled to receive alirocumab and cemiplimab. Fifty-eight patients were eligible per study protocol. Detailed patient characteristics and demographics are described in **Table 1**. All patients had disease progression on prior immune checkpoint blockade alone or in combination with chemotherapies and/or anti-VEGF therapies. Study participants were treated per protocol with cemiplimab 350 mg IV every 3 weeks and alirocumab 150 mg subcutaneously every 2 weeks and according to the study design (**Extended Data Fig. 1)**. Patient disposition is summarized in the CONSORT diagram (**Extended Data Fig. 2).** The clinical results presented from this study are based on the clinical database lock date of April 6, 2026.

### Efficacy in the study population

**Figure 1:**
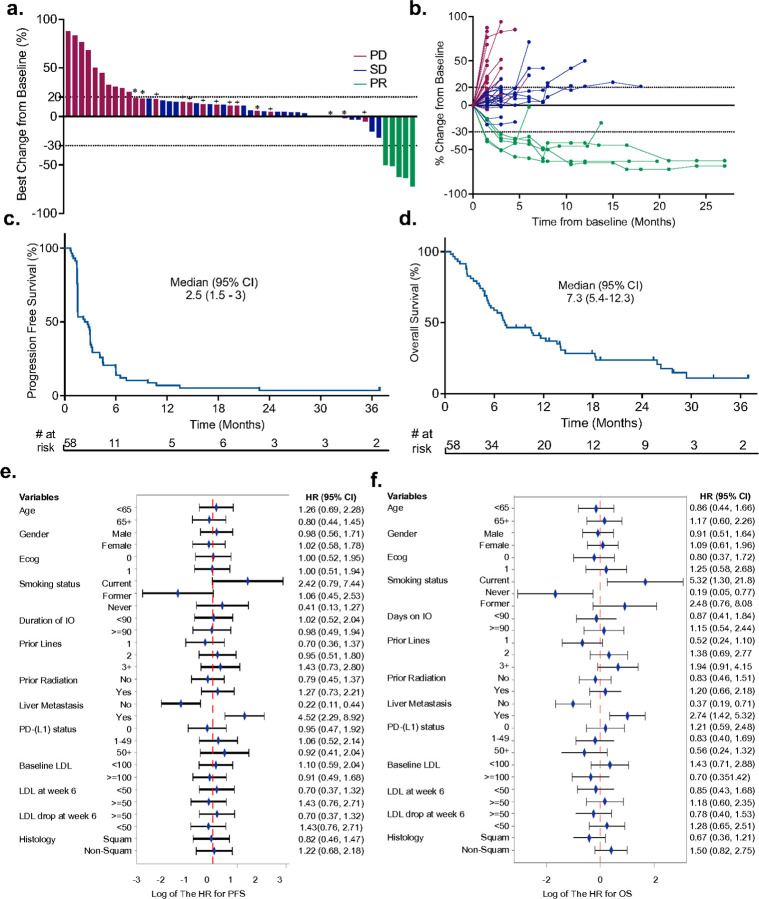
Efficacy of cemiplimab plus alirocumab in the study population. a, Waterfall plot of the best change in target lesions (n=52), ❖ indicates unequivocal progression outside of target lesions, + indicates appearance of new lesions, b, Spider plot illustrating the depth and duration of response from baseline by (n= 52) c, Kaplan-Meier curve of PFS estimate in all patients eligible for efficacy analysis (n=58). d, Kaplan-Meier curve of OS estimate in all patients eligible for efficacy analysis (n=58) e, Forest Plot for PFS in the study population (n=58). f, Forest Plot for OS in the study population (n=58). Six patients had no tumor measurements after baseline scans and were included in the denominator for ORR calculation.

The median follow up was 30.2 months (95% CI, 17.9 – NR). Accounting for the two-stage design the uniformly minimum unbiased estimate of ORR in the study population was 14.98%^27^ (Jennison-Turnbull 90% CI, 5.45, 25.52^28^, one sided *P*-value = 0.29^29^) for the multistage design. An objective response in the study population was achieved in 8.6% (5/58) of study participants. Approximately 50 % (n=29/58) of patients had stable disease as their best response by RECIST, while progressive disease was the best overall response in 41.4% (n=24/58) of patients, resulting in a disease control rate (DCR) of 58.6% (Fig. 1a).The timing of objective response with the corresponding duration of objective response for trial participants are shown in Fig. 1b. The median duration of response was not estimable due to the small number of responses in the study population. At data cut off, objective responses were ongoing in two patients who have been in response for over two years. The other responders progressed at approximately 6 months, 13 months and 20 months (Fig. 1b). The median PFS in the study population was 2.5 months (95% CI, 1.5 – 3.0) (Fig. 1c). The median OS was 7.3 months (95% CI, 5.4 – 12.3) (Fig. 1d). The Kaplan Meier (KM) estimates of the OS rates at 6 and 12 months are 58% and 39%, respectively.

Further analysis by histologic subgroups indicated that 80% (4/5) of responding tumors were of squamous histology, and 20% (1/5) were of non-squamous histology **(Extended Data Fig. 3a-b**). The ORR in the squamous cohort was 12.5% and 3.8% for non-squamous NSCLC. The duration and depth of response by histology is shown in **Extended Data Fig. 3c-d.** When assessed by histology, the median PFS were 1.5 months (95% CI, 1.46 – 3.0), and 2.98 months (95% CI, 1.8 – 4.1) for the squamous and non-squamous cohorts, respectively (**Extended Data Fig. 3e**). There was a trend towards improvement in PFS in squamous NSCLC, but it was not statistically significant (HR 0.74, 95% CI, 0.4–1.3, *P*-value = 0.28). The median OS was 13.64 months (95% CI, 4.28 – 18.25) in squamous NSCLC and 6.1 months (95% CI, 4.82 – 10.46) in non-squamous NSCLC **(Extended Data Fig. 3f)**. However, the OS trend in favor of squamous NSCLC was not statistically significant (HR 0.64, 95% CI 0.34–1.19, *P*-value = 0.17).

We performed a series of exploratory analyses of several clinical characteristics of the study participants. Of all clinical variables, only the presence of liver metastasis was significantly associated with poorer PFS and OS (Fig. 1e–f) by univariate cox proportional model. Current smoking status was significantly associated with poorer OS, and a trend towards poorer PFS. There was a trend toward improved OS for squamous histology was not statistically significant (Fig. 1e–f)

### Safety

All patients who received at least one dose of the study drugs were included in the safety analysis (N=60). In the stage 1 safety lead-in cohort, no dose limiting toxicities were observed allowing for the study to proceed to stage 2.All adverse events (AEs) that were at least possibly treatment related (TRAE) per investigator assessment are summarized in **Table 2** and were predominantly grade 1 or 2. These events were of grade 3 or greater in 12% of patients. There were no dose reductions or treatment related deaths. Treatment discontinuation because of adverse events occurred in two patients (3%). One patient was diagnosed with Guillain-Barre Syndrome on study requiring hospitalization. Another patient had grade 3 transaminitis resulting in treatment discontinuation and remained in response over 24 months. No TRAE of greater than 30% frequency was observed. Anemia was the most common TRAE and was reported in 22% of patients, predominantly grade 2 or less. Liver transaminase elevations and fatigue were the next most common TRAEs, seen in approximately 13% of patients. Arthralgia and rash TRAE were both reported in 8% of patients. Other TRAEs occurred in less than 5% of patients and are described in detail in **Extended Table 1**.

### Biomarker Analysis

#### Clinical and molecular features of the responders.

The durable responses observed in the patients whose tumors achieved an objective response suggests a subset of patients benefitted from the PSCK9 inhibition. However, given the modest response rates in the intent to treat patient population we sought to further understand the specific factors underlying clinical responses. Key clinical characteristics of the responders are summarized in **Extended Table 2**. We examined the genomic profiles of the responding tumors using available next generation sequencing (NGS) results from clinical platforms such as Foundation Medicine, Guardant 360, and Caris. Tumor genomic data was available for 95% (56/58) of the study population, including all the responders **(Extended Table 3)**. Intriguingly, one unifying molecular feature in all responders was the presence of alterations in the PI3K pathway, specifically *PIK3CA, PTEN*, or *AKT1* regardless of histology. The potential significance of this finding became apparent because of the literature implicating AKT1 in regulating SREBP1, one of the transcriptional activators of *PCSK9*^30–32^. Although it has been demonstrated that AKT1 regulates SREBP1, an association between the intratumoral PIK3CA-PTEN-AKT1 axis and PCSK9 expression was not previously known. Therefore, we hypothesized that oncogenic alterations in *PIK3CA, PTEN* or *AKT1* in NSCLC tumors also may increase intratumoral PCSK9 via AKT1 activity on SREBP1, potentially explaining the reversal of immunotherapy resistance seen in these patients upon administration of the PCSK9 inhibitor alirocumab with cemiplimab.

#### Clinical efficacy is higher in *PIK3CA, PTEN* or *AKT1* altered tumors.

**Figure 2.**
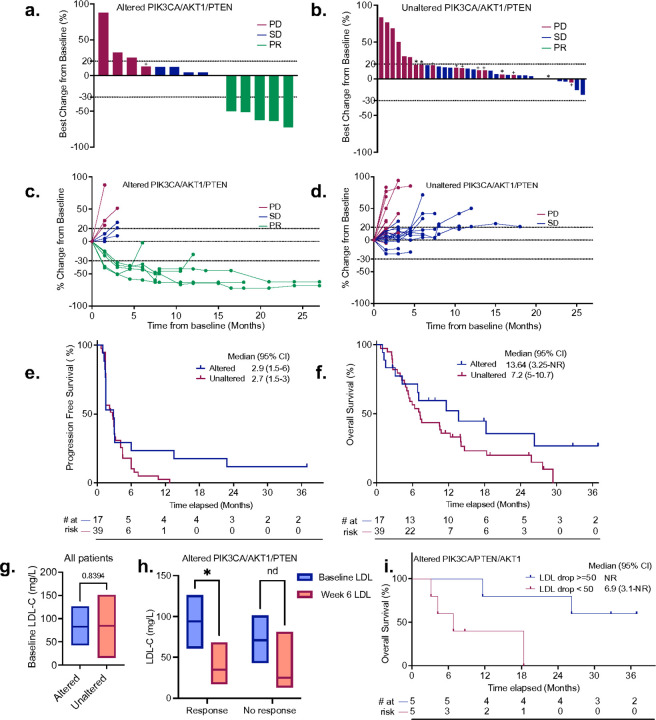
Efficacy evaluation by PIK3CA, AKT1 or PTEN alteration status. **a,** Waterfall plot of the best change in target lesions in PIK3CA./AKT1/PTEN altered patient cohort (n=14). **b,** Waterfall plot of the best change in target lesions in PIK3CA/AKT1/PTEN unaltered patients (n=36). **c,** Spider plot illustrating the depth and duration of response in the PIK3CA/AKT1/PTEN altered cohort (n = 14). **d,** Spider plot illustrating the depth and duration of response in the PIK3CA/AKT1/PTEN unaltered cohort (n = 36). **e,** Kaplan-Meier curve of PFS estimate by alteration status (n=17). **f,** Kaplan-Meier curve of OS estimate by alteration status (n=39). **g,** Floating bars of LDL-C at baseline altered vs unaltered cohorts, **h,** Floating bars comparing paired LDL -C at baseline and 6 weeks in the altered cohort between responders and non-responder, (n=10). **i,** Kaplan-Meier curve of OS estimates in the altered patient cohort by LDL response to alirocumab greater than or less than 50mg/L at 6 weeks (n=10). Three patients each from altered and unaltered cohorts had no subsequent tumor measurements after baseline scan but were included in the denominator of response calculations, PFS and OS analysis. PD, progressive disease; SD, stable disease; PR, partial response. * indicates unequivocal progression outside of target lesion, + indicates appearance of new lesions. Multiple paired t-test with 1% false discovery rate, * positive discovery (g 0.000470); nd, no discovery (g 0.011)

To further evaluate the significance of the *PIK3CA/PTEN/AKT1* axis to the clinical response in the study population, we next performed additional exploratory analyses to determine if efficacy results varied by the presence or absence of these alterations. *PIK3CA/PTEN/AKT1* alterations were present in 28.6% (16/56) of the study population excluding the two patients without tumor genomic data, consistent with expected rates based on TCGA and published data (**Extended Data Fig. 4a-b**),^33–37^. The ORR to the combination of alirocumab and cemiplimab in the *PIK3CA/PTEN/AKT1* altered cohort was 29.4% (5/17) (Fig. 2a). No objective responses were observed in any patient without *PIK3CA, PTEN* or *AKT1* alterations (0/39) (Fig. 2b). The presence of an alteration in *PIK3CA*, *AKT1* or *PTEN* was significantly associated with an objective response, *P*=0.0084 (two-sided, Fisher’s exact). The duration of response ranged from 6 months to over 30 months in the altered group (Fig. 2c–d). The Kaplan Meier estimates of the median PFS were 2.9 months (95% CI, 1.46 – 6.0) in the altered group and 2.7 months (95% CI, 1.5 – 3.0) in the unaltered group (Fig. 2e). The median OS in the altered group was 13.64 months (95% CI, 3.25 – NR) and 7.2 months (95% CI, 5 –10.7) in the unaltered group (Fig. 2f).

Because it has been demonstrated that AKT1 upregulates SREBP1, a transcriptional activator of PCSK9, which in turn can lead to increased LDL-C in cholesterol metabolism, we sought to determine if LDL-C response to the treatment was different in the *PIK3CA/AKT1/PTEN* altered NSCLC compared to the unaltered NSCLC. The median baseline LDL-C did not significantly differ by *PIK3CA/AKT1/PTEN* alteration status. Median baseline LDL-C was 87 mg/dL in altered (range 43–126) and 86 mg/dL (range 16–151) in unaltered patients (Fig 2g). Within the cohort of patients with *PIK3CA*/*PTEN*/*AKT1* alterations, a multiple paired t-test to compare the LDL-C response to alirocumab across responders and nonresponders indicated that LDL-C was significantly changed at 6 weeks in patients who achieved an objective response in contrast to patients with *PIK3CA*/*PTEN*/*AKT1* alterations but without an objective tumor response (Fig. 2h). To control for false positives for the multiple paired analysis, the False Discovery Rate (FDR) was controlled at Q= 1% using the Benjaminini, Krieger, and Yekuleti two-stage step-up method. The change in LDL-C at 6 weeks was higher in the responders (M=56.4, SD=5.4), compared to the nonresponders (M=32.2, SD = 8.8), t (10.5), q= 0.00047, d = 4. This result may suggest a less potent effect of PCSK9 inhibition in the nonresponders with *PIK3CA/PTEN/AKT1* alterations. A univariate unstratified Cox model analysis also demonstrated that the magnitude of reduction in LDL-C at 6 weeks was associated with survival in the *PIK3CA/PTEN/AKT1* altered cohort (Fig. 2i). The median OS in patients who achieved a reduction in plasma LDL-C of 50mg/L or greater at 6 weeks from baseline values was NR and was 6.8 months (3.25-NR) in those with less than 50mg/L reduction in LDL-C. These findings suggest that the anti-tumor response to PCSK9 inhibition may align with its biological role in cholesterol metabolism, however due to the exploratory nature of these analyses and the small sample sizes these results must be interpreted cautiously.

#### Analysis of TCGA demonstrates a relationship between PIK3CA, PTEN, AKT1 and the tumor expression of PCSK9 in NSCLC cohorts.

**Figure 3.**
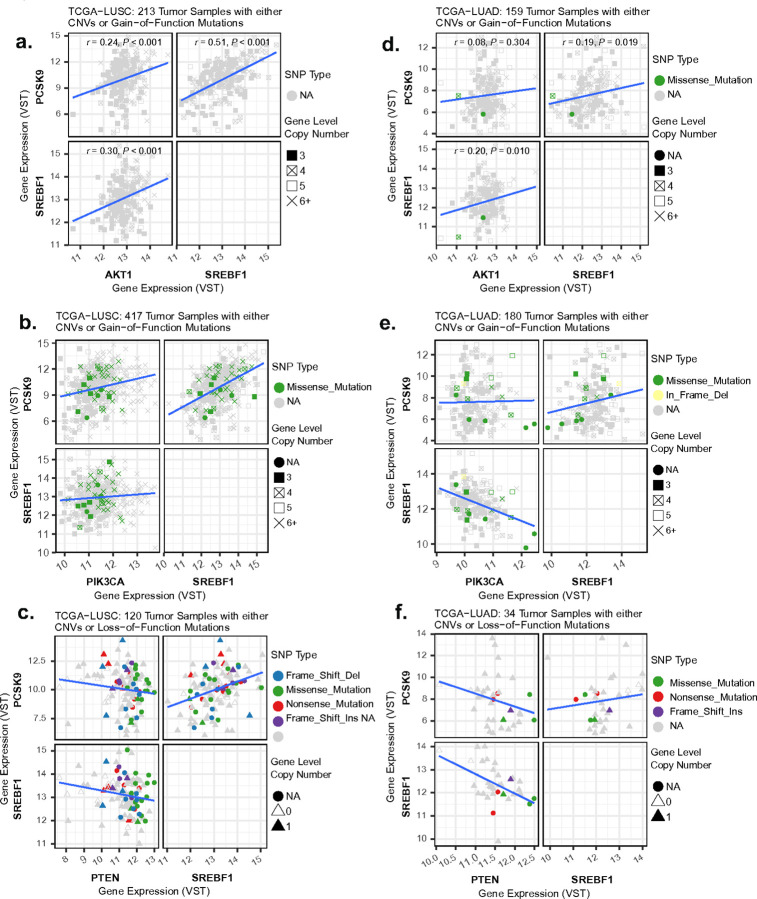
Correlation of PIK3CA, AKT1 or PTEN with PCSK9 expression in TCGA. **a**, Correlation of AKT1 gene expression with PCSK9 and SREBP1 in the Lung Squamous Carcinoma (LUSC) The Cancer Genome Atlas (TCGA) cohort with either AKT1 copy number variants (CNV) or Gain-of-Function Mutations (n=213). **b**, Correlation of PIK3CA gene expression with PCSK9 and SREBP1 in the LUSC TCGA cohort with either PIK3CA CNV or Gain-of-Function Mutations (n=417). **c**, Correlation of PTEN gene expression with PCSK9 and SREBP1 in the LUSC TCGA cohort with either PTEN CNV or Loss-of-Function mutations (n=120). **d**, Correlation of AKT1 gene expression with PCSK9 and SREBP1 in the Lung Adenocarcinoma (LUAD) cohort of TCGA, with either AKT1 CNV or Gain-of-Function Mutations (n=159). **e**, Correlation of PIK3CA gene expression with PCSK9 and SREBP1 in the LUAD cohort of TCGA with either PIK3CA CNV or Gain-of-Function Mutations (n=180). **f,** Correlation of PTEN gene expression with PCSK9 and SREBP1 in the LUAD cohort of TCGA with either PTEN CNV or Loss-of-Function mutations fn=34)

To test the association between PIK3CA, PTEN or AKT1 with PCSK9, we first performed analysis of The Cancer Genome Atlas (TCGA) lung adenocarcinoma (LUAD) and lung squamous (LUSC) cohorts. *PIK3CA/PTEN/AKT1* alterations were more prevalent in squamous compared to non-squamous NSCLC **(Extended Data Figure 4a-b**), consistent with previous reports^33–37^. The gene expression of *PIK3CA*, *PTEN* and *AKT1* in tumor samples with either copy number variants (CNV), *PIK3CA* gain-of-function (GOF) mutations, *AKT1* (GOF) mutations or *PTEN* loss of function (LOF) mutations were correlated with the expression of *PCSK9* and *SREBF1* (encodingSREBP1)genes. Specifically, in the 213 LUSC tumor samples with *AKT1* CNVs or GOF mutations, *AKT1* expression was positively correlated with both *PCSK9* expression (Pearson’s r = 0.24; *P*-value<0.001) and *SREBF1* expression (r = 0.30; *P*-value<0.001) (Fig. 3a). Similarly, in the 417 LUSC tumor samples with CNVs or GOF *PIK3CA* mutations, *PIK3CA* expression correlated positively with *PCSK9* expression (r = 0.23; *P*-value<0.001) and trended towards a positive correlation with *SREBF1* (r = 0.09; *P*-value =0.073) (Fig. 3b). In the 120 LUSC tumor samples with PTEN CNVs or LOF mutations, *PTEN* gene expression correlated negatively with *PCSK9* (r=−0.13; *P*-value = *0.14)* and *SREBF1* expression (r = −0.18; *P*-value = 0.047), suggesting higher *PCSK9* expression in *PTEN*-deleted tumors(Fig. 3c). *SREBF1* expression consistently correlated positively with *PCSK9* (r = 0.51, 0.46, and 0.34, respectively; all *P*-value <0.001) across all analyses in LUSC (Fig. 3a–c). A similar but less robust trend between the expression of *PIK3CA, PTEN*, or *AKT1* and *PCSK9/SREBP1* was observed in the LUAD cohort (Fig. 3d–f). Additional correlation of the RNA similarly demonstrated a statistically significant correlation between the PIK3CA/PTEN/AKT1 and SREBP1/PCSK9 axis (**Extended Figures 5a-b**). Moreover, the RNA expression of these alterations correlated to their protein expression in matched cohorts LUSC and LUAD cohorts of the TCGA (**Extended Figure 6a-b)** These findings provide additional evidence supporting further investigation into the potential role of *PIK3CA/AKT1/PTEN*, key players of the PI3K pathway, in regulating *PCSK9* in tumors.

#### Inhibition of PIK3CA and AKT1 attenuates tumor cell secreted PCSK9 while knockdown of PTEN enhances PCSK9 secretion *in vitro*.

**Figure 4.**
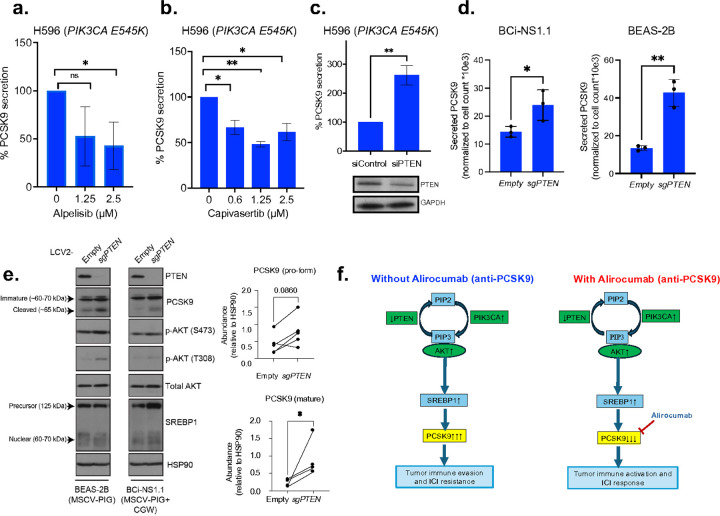
Manipulation of PIK3CA, AKT1 or PTEN regulates tumor secreted PCSK9 *in-vitro*. **a,** The adeno-squamous NSCLC cell line H596 (*PIK3CA E545K)* was treated with alpelisb (PIK3CA inhibitor) at the indicated doses for 24 hours and the supernatants were subjected to PCSK9 ELISA. The graph shows the percent reduction in secreted PCSK9 relative to untreated control in 3 independent replicates +/−SD; **b,** The adeno-squamous NSCLC cell line H596 (*PIK3CA E545K)* was treated with capivasertib (AKT1 inhibitor) at the indicated doses for 24 hours and the supernatants were subjected to PCSK9 ELISA. The graph shows the percent reduction in secreted PCSK9 relative to untreated control in 3 independent replicates+/−SD **c, top,** the adeno-squamous NSCLC cell line H596 (*PIK3CA E545K)* was transfected with control or PTEN-specific siRNA, and the supernatants were subjected to PCSK9 ELISA. The graph shows the percent increase in secreted PCSK9 relative to untreated control in 3 independent replicates +/−SD; **bottom,** Corresponding cell lysates from the H596 (*PIK3CA E545K)* with control siRNA or the PTEN-specific siRNA were immunoblotted with the indicated antibodies to confirm PTEN knockdown; shown is a representative immunoblot demonstrating knockdown of PTEN relative to control, d, Representative ELISA results from BEAS-2B or BCi-NS1.1 basal/epithelial cell lines +/− sgPTEN; **e** Cell lysates from BEAS-2B or BQ-NS1.1 basal/epithelial cell lines +/− sgPTEN were immunoblotted with the indicated antibodies, **f,** Proposed pathway of response. All graphs show average of at least 3 biological replicates. Student’s unpaired t-test, ns not significant p>0.05, * p <0.05, ** p<0.01, *** p <0.001, **** p<0.0001

Next, we evaluated the impact of manipulating PIK3CA, PTEN or AKT1 on PCSK9 expression *in vitro*. The human adeno-squamous NSCLC cell line H596 (*PIK3CA E545K*) was treated with increasing doses of the PIK3CA inhibitor, alpelisib for 24 hours followed by ELISA of the supernatant to determine whether inhibition of PIK3CA would impact PCSK9 secretion. Consistent with our hypothesis that PIK3CA activity increases intratumoral PCSK9, pharmacologic inhibition of PIK3CA decreased tumor cell secreted PCSK9 in a dose dependent manner (Fig. 4a). H596 (*PIK3CA E545K*) cells were also subjected to AKT1 inhibition using capivasertib. Cells were treated with increasing concentrations of capivasertib for 24 hours and PCSK9 was measured in the supernatant by ELISA. AKT1 inhibition by capivasertib also suppressed tumor cell secreted PCSK9 (Fig. 4b). To evaluate the contribution of PTEN, *PTEN* was knocked down via siRNA in the H596 (*PIK3CA E545K*) cells and PCSK9 was measured in the supernatant by ELISA. *PTEN* siRNA led to an increase in cell-secreted PCSK9 when compared to control siRNA cells (Fig. 4c).

Furthermore, stable CRISPR-mediated knockout of *PTEN* in two normal human lung basal/epithelial-like cells, BEAS-2B and BCi-NS1.1 cells demonstrated significant increase in PCSK9 secretion in the PTEN knockout compared to empty vector controls, as detected by ELISA (Fig. 4d). There was also a modest but significant increase in mature PCSK9 and a subtle induction of phospho-AKT1 and nuclear SREBP1 in the PTEN knockout as determined by immunoblot (Fig. 4e), although it was not strikingly different in *PTEN* knockout cells relative to controls. In contrast, in a panel of two distinct human NSCLC cell lines (H226 and h2703) that lack existing alterations in the *PIK3CA/PTEN/AKT1* pathway but have basal AKT1 and PCSK9 activity, inhibition of AKT1 via capivasertib did not significantly alter PCSK9 assessed by immunoblot or ELISA after treatment for 14 or 24 hours (**Extended Fig. 7b**), suggesting the lack of benefit in the absence of pathway activation and/or low basal PCSK9 expression.

Collectively, the data suggest a model whereby activation of PIK3CA or AKT1 and/or inactivation of PTEN in NSCLC tumors or normal basal cells can lead to increased cell secretion of PCSK9, possibly via SREBP1 to represent an upstream mechanism of PCSK9 regulation in NSCLC tumors. Consequently, PSCK9 may promote immune evasion, thus explaining the responses seen in tumors harboring *PIK3CA, PTEN* and *AKT1* alterations when treated with alirocumab (Fig. 4f). These findings suggest that these alterations could potentially function as molecular biomarkers of response to PCSK9 inhibition. Taken together, these findings suggest that the response to PCSK9 inhibition in NSCLC with progression on prior ICI-based therapy could be dependent on the alteration status of *PIK3CA, AKT1* or *PTEN* in the tumors, which tends to be enriched in squamous NSCLC compared to non-squamous NSCLC^34–38^. Amongst patients with *PIK3CA, PTEN* or *AKT1* alterations, the magnitude of LDL response to anti-PCSK9 therapy could be an indication of anti-tumor efficacy.

## Discussion

We herein report the results of a clinical trial evaluating the efficacy and safety of anti-PCSK9 directed therapy in combination with immune checkpoint blockade to overcome immunotherapy refractoriness in NSCLC patients. In this previously treated metastatic NSCLC patient population who had progressed on prior anti-PD1/PD-L1-based therapies, the addition of the PCSK9 inhibitor, alirocumab, to the anti-PD1 antibody, cemiplimab, was well tolerated and reversed immunotherapy resistance in a small proportion of patients. Although the clinical outcomes in the overall population compare favorably to many 2^nd^ and 3^rd^ line standard of care options, and immunotherapy retrial studies, we identified a subset of patients with *PIK3CA, PTEN* or *AKT1* alterations that are likely to obtain superior response and survival benefits. Our translational studies provide a biological rationale for the observed response pattern.

We report for the first time clinical responsiveness to the combination of PCSK9 inhibition and checkpoint blockade in *PIK3CA, PTEN* and *AKT1* altered NSCLC in the immunorefractory setting. Multiple studies demonstrated that PI3K pathway activation drives immunotherapy resistance through several mechanisms including decreased T cell infiltration and trafficking, increased release of immunosuppressive cytokines, upregulation of immune checkpoint ligands and fostering a suppressive remodeling of the immune microenvironment by enriching for MDSCs and TAMs^38–42^, but none in relationship to tumor cell PCSK9 production. Interestingly, similar mechanisms were implicated in PCSK9 mediated immune resistance, however the mechanisms by which tumor cells produce PCSK9 were not previously known. Our findings not only put forward a safe and feasible therapeutic strategy to overcome immunotherapy resistance in a subset of NSCLC tumors but also shed light on one of possibly many mechanisms promoting PCSK9-mediated immunotherapy resistance in NSCLC.

The results suggest that tumor intrinsic PCSK9 activation is mediated at least in part by the *PIK3CA/PTEN/AKT1* axis. PIK3CA activates the phosphorylation of phosphatidyl-inositol phosphate (PIP) 2 to PIP3 to induce phospho-AKT1 (pAKT1) kinase activity. AKT1 activation secondary to upstream PIK3CA signaling or in the presence of activating AKT1 mutations and amplifications are reported to enhance SREBP1 activity leading to increased PCSK9^43–47^. Therefore, tumor cells harboring these alterations are likely to have increased PCSK9 production via the same mechanism. The tumor suppressor PTEN is a negative regulator of PIK3CA activity, inhibiting the phosphorylation of PIP2 to PIP3; therefore, PTEN downregulation can result in unopposed pAKT1 activity. We predict that in the context of *PIK3CA* oncogenic alterations (via mutation or amplification), *PTEN* loss or deletion, or activating *AKT1* mutations or amplification, one downstream effect is increased intratumoral PCSK9 that contributes to immune evasion based on previously described mechanisms (Fig. 4f). Our pharmacologic and genetic inhibition of these pathways provide preclinical evidence supporting their impact on PCSK9 regulation. Although the association between PI3K/AKT1 pathway and SREBP1 regulation was previously published, and the regulation of PCSK9 by SREBP1 was previously known, here we identify a previously unreported link between oncogenic intratumoral *PIK3CA/PTEN/AKT1* signaling and PCSK9 regulation that could mediate immunotherapy resistance in NSCLC and explain the response pattern seen in the presence of these alterations.

The durable responses and clinical benefit demonstrated in the *PIK3CA/PTEN/AKT1* altered patient subsets suggest that *PIK3CA/PTEN/AKT1* alterations could serve as biomarkers of response to guide patient selection for anti-PCSK9 based therapies. The clinical trial response rates are reminiscent of initial trials of targeted therapies such as EGFR and NTRK tyrosine kinase inhibitors that underscored our understanding and current treatment paradigm of these actionable genomic alterations. Although we identified a small subset of patients likely to benefit from anti-PCSK9 based combination with checkpoint blockade, most treatments for NSCLC are highly efficacious in small subsets of patients.

A limitation of the current study is the limited number of patients. It is also important to note that about 20% of non-responders had *PIK3CA/PTEN/AKT1* alterations. The reason for the lack of response in these patients is currently unclear. However, some important considerations are noteworthy. First, the regulation of PCSK9 is complex, involving other genes that are not routinely captured on clinically utilized NGS panels in lung cancer. Therefore, the presence of genomic alterations in other upstream regulators of SREBP1 and PCSK9 that could abrogate the impact of *PIK3CA/PTEN/AKT1* alterations on PCSK9 is possible but was not investigated in the current study. Furthermore, the downstream impact of PCSK9 on the immune response is an evolving area of active investigation. Although it would be important to determine the immune cell populations in the tumors of responders and nonresponders, such analyses are not possible in the current study due to lack of tissue availability. We have initiated a clinical trial adding PCSK9 inhibition to neoadjuvant therapy in NSCLC. The post-surgical samples will provide ample specimen for comparisons across patients treated with and without alirocumab to determine the impact of PCSK9 and PCSK9 inhibition on the tumor immune microenvironment and an in-depth investigation of the molecular signaling pathways at play.

In conclusion, we report the result of the first clinical investigation of the combination of a PCSK9 inhibitory antibody with anti-PD1 antibody in patients with metastatic immune-refractory NSCLC. We discovered a relationship between *PIK3CA, PTEN* and *AKT1* alterations and the anti-tumor response to anti-PCSK9 and anti-PD1 antibody. The presence of these alterations yielded a clinically meaningful ORR of approximately 30% with survival benefits. As a potential biomarker of response, the testing for *PIK3CA/PTEN/AKT1* alterations is reproducible and accurate by currently FDA approved NGS assays. Furthermore, our bioinformatic analyses and *in vitro* testing bolster the existing literature in support of these alterations as biologically valid markers of response to anti-PCSK9 antibody. Given the limited number of patients in this study, these findings warrant further investigation and validation in a larger, confirmatory trial.

## Methods

### Trial Design

TOP 2201 is a phase II, single arm, open label trial designed to evaluate the clinical activity and safety of alirocumab, an anti-PCSK9 monoclonal antibody, in combination with cemiplimab, an anti-PD1 monoclonal antibody in patients with metastatic non-small cell lung cancer (NSCLC). Eligible patients had metastatic non-small cell lung cancer (NSCLC) with disease progression on or after prior immune checkpoint blockade alone or in combination with chemotherapy and/or anti-VEGF therapeutics. The primary objective was to determine the clinical activity of the combination as measured by response rate. A secondary objective was to evaluate the safety and tolerability of the combination. Secondary efficacy endpoints included progression-free survival (PFS), overall survival (OS), duration of responses (DOR) and disease control rates (DCR). An exploratory objective was to evaluate the pharmacodynamic change in LDL cholesterol levels measured in serial plasma collection while on treatment with alirocumab and molecular markers of response. A discussion of the design and preclinical rationale was previously published^47^.

The trial was designed in two stages. In the stage 1 cohort, a safety lead-in and efficacy evaluation were planned for the first 15 patients. Patients received two doses of cemiplimab 350 mg IV every three weeks along with 3 doses of alirocumab 150 mg subcutaneously every two weeks followed by evaluation. The prespecified protocol requirements in order to proceed from stage 1 to stage 2 were (1) at most 2 dose limiting toxicities (DLTs) in the first 10 patients and (2) at least two responses to be achieved in the first 15 patients in the stage 1 cohort. DLTs were collected for the first 10 patients only. Toxicity was graded according to the NCI CTCAE version 5.0 criteria. Responses were assessed by Response Evaluation Criteria in Solid Tumors version (RECIST) v1.1. A subject was considered evaluable for DLT if they received at least one dose of cemiplimab and alirocumab. In the stage 2 expansion cohort, patients received the same regimen of cemiplimab 350mg IV every 3 weeks with alirocumab 150mg subcutaneously every 2 weeks until disease progression or unacceptable toxicities, completion of 24 months of study treatment or other reasons for subject discontinuation as indicated in **Appendix 1.** Toxicity related dose modifications were not allowed; however, treatment delays were allowed for immune mediated adverse events. Each cycle was 6 weeks long, allowing for 2 administrations of cemiplimab infusion and 3 injections of alirocumab.

Treatment assessments included radiographic images (CT and/or MRI scans) at baseline and every 6 weeks for the first year, and then every 12 weeks for the second year. Standard lab tests including CBC and CMP were required at baseline and every 3 weeks prior to administration of cemiplimab. Thyroid profile was required every 6 weeks. LDL cholesterol was obtained at baseline, with the first restaging at 6 weeks of treatment. Adverse events including seriousness, grade and relationship to study drug were documented using the NCI-CTCAE version 5.0. Treatment beyond unconfirmed progressive disease (UPD) was allowed as long as patients were clinically stable; deemed to have clinical benefit without rapid disease progression; tolerating study treatment; and treatment beyond UPD did not delay an imminent intervention to prevent serious complications of disease progression. If a patient was treated beyond UPD, confirmatory scans were obtained 6 weeks after initial UPD scans.

### Patients

Patients with histologically and/or cytologically confirmed metastatic stage IV NSCLC were eligible. All patients had to have progressed after prior anti-PD-1/PDL1 directed therapy, either alone or in combination with other agents such as chemotherapy, anti-CTLA4 or anti-VEGF. Patients with actionable molecular targets including EGFR, ALK, ROS1, MET exon 14, RET, BRAF, NTRK were allowed, but must have had progression on prior targeted therapy. There were no limits to the number of prior lines of therapy. Exclusion criteria included prior treatment with PCSK9 inhibitors, known auto-immune conditions requiring systemic immune suppressive therapy other than 10mg or less of prednisone, receiving chronic systemic treatment with corticosteroids or another immunosuppressive agent, interstitial pneumonitis from any cause, severe COPD or pulmonary disease with hypoxemia, symptomatic brain or leptomeningeal disease, uncontrolled infection, intolerance to prior PD-1/L1 treatment including discontinuation for severe or recurrent toxicities. A full list of inclusion and exclusion criteria are provided in the protocol (**Appendix 2**).After an interim analysis of the first 40 patients, the protocol was amended to restrict enrollment to squamous NSCLC patients due to initial preliminary data^49^.

### Clinical Trial End points

The primary endpoint was the objective response rate associated with the addition of alirocumab to cemiplimab as assessed by RECIST 1.1. Response rate was defined as the proportion of patients with complete response (CR) or partial response (PR) per RECIST 1.1 criteria. All eligible patients (n=58) who received at least one dose of alirocumab and cemiplimab were considered for the primary endpoint analysis.

The secondary endpoints were safety; disease control rate (defined as proportion of patients with stable disease (SD), PR, or CR); progression-free survival (defined as the time from the first dose of treatment to the time of progressive disease or death in all patients eligible for efficacy analysis); overall survival and duration of response (time from first treatment to the time of progressive disease or death among patients who have a documented treatment response). All patients who received at least one dose of study treatment were included in the safety analysis (n = 60) Exploratory endpoints included the mean/median plasma LDL cholesterol levels at baseline and changes from baseline treatment levels and molecular markers of response or lack thereof.

### Statistical Analysis

Patient demographics and baseline disease characteristics were summarized using descriptive statistics. Mean, median, standard deviation minimum and maximum were used for continuous variables. Categorical variables were reported by frequency and the corresponding percentages.Time-to-event secondary endpoints including PFS, OS and DOR were estimated by the Kaplan-Meier method. Exploratory subgroup analyses were performed using the univariate cox model. A Fisher’s exact test was used when comparing differences in rates by groups. Spider and waterfall plots were used to depict the change in tumor response over time. To control for false positives, in LDL-C comparison within PIK3CA/AKT1/PTEN altered NSCLC, the False Discovery Rate (FDR) was controlled at Q= 0.01 using the Benjamini, Krieger-Yekutleli two-step linear method. For response rate and disease control rate, counts and percentages were calculated and compared between mutation status using the Fisher’s exact test. There were no adjustments for multiple testing due to the exploratory nature of these analyses. The above statistical analyses were analyzed using GraphPad Prism (Version 11.0.1 (99). An independent statistical review and verification was performed using the SAS software (version 9.4, SAS Institute Inc.).

### Bioinformatics Considerations for the Analysis of TCGA Data

#### Data cohorts:

The analysis population consisted of cohorts of lung adenocarcinoma (LUAD)^50^ and lung squamous cell carcinoma (LUSC)^51^ from The Cancer Genome Atlas (TCGA) database. RNA-seq count data, Reverse Phase Protein Array (RPPA) data, and Single nucleotide variation (SNV) and gene-level copy number variation (CNV) data were all obtained from the Genomic Data Commons (GDC)^52^ data portal using the R package TCGAbiolinks (v2.30.0)^53^. The gene alteration types were summarized at level of all available patients. The analyses were limited to patients with relevant clinical annotations. For LUAD, only primary solid tumor samples from patients primarily diagnosed as “Adenocarcinoma, NOS” were included. For LUSC, only primary solid tumor samples from patients primarily diagnosed as “Squamous cell carcinoma, NOS” were included.

#### RNA-seq expression correlation analysis integrated with genomic alterations:

RNA data provided in raw gene counts were normalized and log2-transformed using the variance-stabilizing transformation (VST)^54^ method implemented in the R package DESeq2 (v1.26.0)^55^ SNV data provided in Mutation Annotation Format (MAF) were processed and summarized using R package maftools (v2.18.0)^53^. Mutation effect annotations specifically linked to the SNV events detected in PTEN, PIK3CA and AKT1 were obtained from the OncoKB^56^ database using the R package oncokbR (v0.0.0.9001, http://www.karissawhiting.com/oncokbR). The database uses the annotation categories “Unknown”, “Inconclusive”, “Likely Loss-of-function”, “Loss-of-function”, “Likely Neutral”, “Neutral”, “Likely Gain-of-function”, or “Gain-of-function”. To these, we added an additional “Not Detected” category which was assigned to samples with no mutation detected in the gene. For some downstream analyses, categories were consolidated by merging “Likely Loss-of-function” into “Loss-of-function”, “Likely Gain-of-function” into “Gain-of-function”, and “Likely Neutral” into “Neutral”.CNV data consisted of estimated copy number levels for each gene in each sample, and events were categorized as deletions if copy numbers were 0 or 1 copies, and as amplifications if copy numbers were 3, 4, 5, or ≥6 copies. For each cohort and each gene (*PTEN, PIK3CA, AKT1*), Pearson correlation coefficients were used to assess the following relationships: (i) expression of the gene versus *SREBF1*, (ii) expression of the gene versus *PCSK9*, and (iii) expression of *SREBF1* versus *PCSK9*. These relationships were assessed in the following subsets: (i) all tumor samples with relevant clinical annotations as defined above (ii) tumor samples with either relevant mutation effects in consolidated categories (“Loss-of-function” for *PTEN*; “Gain-of-function” for *PIK3CA* and *AKT1*) or CNV alterations (deletions for *PTEN*; amplifications for *PIK3CA* and *AKT1*). There were no adjustments for multiple testing due to the exploratory nature of these analyses.

#### RNA-seq expression correlation analysis:

TCGA RNA-seq count data were obtained from the Genomic Data Commons (GDC)^53^ data portal using the R package TCGAbiolinks (v2.30.0)^54^. Raw gene counts were normalized and log2-transformed using the variance-stabilizing transformation (VST)^55,56^ method implemented in the R package DESeq2 (v1.26.0)^57^. Within each cohort, Pearson correlation coefficients were used to assess relationships between (i) gene-level expressions of PTEN, PIK3CA, and AKT1 versus SREBF1, and (ii) SREBF1 versus PCSK9.

#### Protein expression correlation analysis:

Normalized protein expression levels were provided on the log2 scale. Within each cohort, Pearson correlation coefficients were used to assess relationships between gene-level expressions of *PTEN*, *PIK3CA*, and *AKT1* versus protein-level expressions of their respective peptide targets (PTEN, PI3KP110ALPHA, and AKT/AKT_pS473/AKT_pT308). There were no adjustments for multiple testing due to the exploratory nature of these analyses.

#### Computing environment:

The analysis of the TCGA data was performed in the R Statistical Environment^58^, using extension packages from the Comprehensive R Archive Network (CRAN; https://cran.r-project.org/) and the Bioconductor project^59^. To ensure reproducibility, R package knitr (v1.45)^60^ was used for generating dynamic reports.

### Human cell lines

Human cell lines included lung adeno-squamous cell line, H596 (ATCC cat# HTB-178), lung squamous carcinoma cell line H226, (ATCC cat# HTB-182), H1703 (ATCC CRL-5889), BEAS-2B (ATCC #CRL-3588; epithelial cells isolated from normal human bronchial epithelium that undergo squamous differentiation with serum) and immortalized basal cell line BCi-NS1.1 generously obtained from Dr. Ronald G Crystal^61^. HEK-293T/17 cells (ATCC cat# CRL-11268) were used to produce lentivirus. All cell lines were tested for mycoplasma every three months and were negative. Cell line identities were confirmed via STR profiling within six months of usage, last performed in July 2024. No commonly misidentified cell lines were used in this study. H596, H1703 and H226 cells were grown in RPMI supplemented with 1% penicillin/streptomycin, with or without 1% L-glutamine, and 10% FBS and passaged or expanded every two days. BEAS-2B and BCi-NS1.1 cell lines were grown in Bronchial Epithelial Growth Media (BEGM, Lonza, CA) supplemented with 0.5% penicillin/streptomycin. BEAS-2B/BCi-NS1.1 cells were passaged or expanded every 2–3 days, using 0.05% trypsin EDTA for 5 min, at 37°C for dissociation, and quenching with the addition of HEPES buffered saline supplemented with 15% fetal bovine serum (FBS). HEK-293T/17 cells were grown in DMEM supplemented with 1% penicillin/streptomycin, 1% L-glutamine, and 10% FBS and passaged or expanded every two days.

### Human cell line infections

BEAS-2B cells were infected with viruses derived from the following constructs: MSCV-Puro-IRES-GFP (PIG) Empty (Addgene Plasmid #21654) or carrying wild-type human MYC, and Lenti-CRISPRv2 (LCV2) with an sgRNA against PTEN (sgPTEN: 5’-GAC TGG GAA TAG TTA CTC CC -3’) in the LCV2-hygro backbone (Addgene Plasmid #98291). In brief, high-titer virus (~1–5 ×107 pfu) was produced using HEK-293T cells transfected with a three-plasmid system including the targeting construct and viral packaging plasmids (pCMV delta R8.2, Addgene Plasmid #8455; pCMV-VSVG, Addgene Plasmid #8454). Viruses were harvested at 48 and 72 h post-transfection, concentrated by ultracentrifugation (25,000 RPM for 1.45 h), resuspended in 1X sterile PBS, and stored at −80°C until use. Cells were plated at ~500,000 cells per well of a 6-well plate in BEGM and incubated overnight to allow them to adhere. The next day, plated cells were subjected to spinoculation at 37°C, 250 × g, for 30 min in 2 mL BEGM, 5 μg/mL polybrene (Santa Cruz cat# sc-134220), and 20 μL high-titer virus. Cells were selected 48 h after spinoculation with hygromycin (for LCV2-hygro-infected cells) and/or puromycin (for PIG-infected cells). Validation of gene editing was confirmed via T7 endonuclease assays (LCV2-infected cells) and/or immunoblotting.

### Immunoblotting

For human cell lines, protein lysates were prepared and quantified (BioRad DC Protein Assay; cat# 5000111), separated via SDS-PAGE, and transferred to PVDF membranes (BioRad cat# 1704157) using a Trans-Blot Turbo Transfer System (BioRad cat# 1704150). Membranes were blocked for 1 h in 5% milk followed by overnight incubation with primary antibodies at 4°C. Membranes were washed for 3x 10 min at room temperature (RT) in TBS-T. Mouse and rabbit HRP-conjugated secondary antibodies (Jackson ImmunoResearch, 1:10,000) were incubated for 1 h in 5% milk at RT followed by washing 3x 10 min at RT in TBS-T. Membranes were exposed to WesternBright HRP Quantum substrate (Advansta cat# K-12045-D50) and detected on Hyblot CL film (Denville Scientific Inc). Primary antibodies included: total AKT (1:1000, CST cat# 9272), PCSK9 (1:1000, CST cat# 85813), phospho-AKT (Ser473) (1:1000, CST cat# 4060S), phospho-AKT (Thr308) (1:1000; CST cat# 13038), PTEN (1:1000; CST cat# 9559), PTEN (1:1000; CST cat# 9552S), SREBP1 (1:1000; CST cat# 95879S), SREBP2 (1:1000; CST cat# 25940S), and HSP90 (1:1000, CST cat# 4877) and GAPDH (1:1000; CST cat # 5174S and CST cat# 2118S) as loading control.

### Drug assays

H596 cells were plated in 96 well plates at 30,000 or 50,000 cells per well and 100ul of growth media 24 hours prior to drug treatment. Capivasertib and alpelisib were added at the indicated doses. After 24 hours, supernatants were collected for ELISA and cells subjected to MTS assay to assess for viability/cell death secondary to inhibitor treatment. BEAS and BCI.c cells were plated in 6-well tissue-culture treated dishes at 1.25 × 10^5 cells per well in 1 mL of growth media 24 h prior to drug treatment. Capivasertib (AKT inhibitor, MedChemExpress cat#HY-15431) was added to cells in 1 mL media for a final concentration of 10 mM/well and cells were incubated for 72 h. Following treatment, cell pellets were harvested (and counted manually), then prepared for western blot analysis. Cell supernatants were frozen at −20 °C for downstream ELISAs.

### siRNA Transfection

H596 cells were plated on Day 0 and transfected 24 hours later at approximately 70–90% confluency utilizing either siRNA against PTEN (Cell Signaling *SignalSilence^®^ PTEN siRNA cat# 6251)* or the corresponding control siRNA (Cell Signaling *SignalSilence^®^ Control siRNA, cat# 6568*). siRNA duplexes were combined with Lipofectamine^™^ RNAiMAX Transfection Reagent (Thermo Fisher Scientific, cat#3117130) according to the manufacturer’s instructions and added directly to the culture media of cells plated in 10-cm tissue culture dishes. Cells were incubated with siRNA-containing media for 24 hours, after which the media was replaced with fresh growth media. Conditioned media were collected 24 hours after media replacement (48 hours after transfection) for PCSK9 ELISA analysis. Efficiency of PTEN knockdown was evaluated by PTEN immunoblot of lysates obtained from the cells harvested at the same time point.

### Enzyme-linked immunosorbent assay (ELISA)

ELISAs were performed using a standard sandwich protocol and the PCSK9 DuoSet ELISA Kit (R&D Systems cat#DY3888) and the DuoSet Ancillary Reagent Kit 2 (R&D Systems cat#DY008B). According to manufacturer’s protocol, 96-well microplates were coated overnight at RT with capture antibody diluted in 1X PBS. Plates were washed three times with wash buffer, blocked with reagent diluent for 1 h, and washed again. Samples and standards diluted in reagent diluent were added in duplicate and incubated for 2 h at room temperature. Following three washes, detection antibody was added for 2 h, plates were washed, and Streptavidin–HRP was applied for 20 min at RT in the dark. After a final wash, substrate solution was added for 20 min before stopping the reaction with stop solution. Absorbance was measured at 450 nm with wavelength correction at 540 or 570 nm using a microplate reader. PCSK9 concentrations were normalized to total cell count per well at the time of collection.

## Supplementary Material

Supplementary Files

This is a list of supplementary files associated with this preprint. Click to download.

- Appendix2.Protocol.pdf
- ExtendedDataFigure6.pdf
- ExtendedTable3.pdf
- ExtendedDataFigure2.CONSORT.pdf
- ExtendedTable2.pdf
- ExtendedTable1.Alladverseeventsatleastpossiblyrelatedtotreatment.pdf
- Table2.SummaryofTRAEs6.3.26.pdf
- ExtendedDataFigure5.pdf
- Table1.pdf
- Appendix1ReasonsforIneligibility.pdf
- ExtendedDataFigure3.pdf
- nrreportingsummaryOduah6.19.2026.pdf
- ExtendedDataFigure4.pdf
- ExtendedDataFigure1.pdf
- ExtendedDataFigure7.pdf

## Data Availability

The source code for reproducing the analysis of the TCGA is available through a public code repository (https://gitlab.oit.duke.edu/dcibioinformatics-internal/projects/antonia-oduah-lung-tcga). The latter also provides the Singularity^51^ definition file for regenerating the computational environment. The datasets supporting the clinical findings of this study are not publicly available due to information that could compromise research participant consent. Individual participant data can be shared upon request to the corresponding author. Source code for reproducing the clinical data statistical analysis is available by contacting the corresponding author.
